# Genome-wide meta-analysis identifies novel loci conferring risk of acne vulgaris

**DOI:** 10.1038/s41431-023-01326-8

**Published:** 2023-03-16

**Authors:** Maris Teder-Laving, Mart Kals, Anu Reigo, Riin Ehin, Telver Objärtel, Mariliis Vaht, Tiit Nikopensius, Andres Metspalu, Külli Kingo

**Affiliations:** 1 Estonian Genome Center, Institute of Genomics, University of Tartu, Tartu, Estonia; 2 Institute for Molecular Medicine Finland (FIMM), HiLIFE, University of Helsinki, Helsinki, Finland; 3 Institute of Health Technologies, Tallinn University of Technology, Tallinn, Estonia; 4 BioCC Ltd, Tartu, Estonia; 5 Institute of Molecular and Cell Biology, University of Tartu, Tartu, Estonia; 6 Faculty of Medicine, Institute of Clinical Medicine, University of Tartu, Tartu, Estonia; 7 Tartu University Hospital, Tartu, Estonia

**Keywords:** Genome-wide association studies, Genetics research

## Abstract

Acne vulgaris is a common chronic skin disorder presenting with comedones, cystic structures forming within the distal hair follicle, and in most cases additionally with inflammatory skin lesions on the face and upper torso. We performed a genome-wide association study and meta-analysis of data from 34,422 individuals with acne and 364,991 controls from three independent European-ancestry cohorts. We replicated 19 previously implicated genome-wide significant risk loci and identified four novel loci [11q12.2 (*FADS2*), 12q21.1 (*LGR5*), 17q25.3 (*FASN*), and 22q12.1 (*ZNRF3-KREMEN1*)], bringing the total number of reported acne risk loci to 50. Our meta-analysis results explain 9.4% of the phenotypic variance of acne. A polygenic model of acne risk variants showed that individuals in the top 5% of the risk percentiles had a 1.62-fold (95% CI 1.47–1.78) increased acne risk relative to individuals with average risk (20–80% on the polygenic risk score distribution). Our findings highlight the Wnt and MAPK pathways as key factors in the genetic predisposition to acne vulgaris, together with the effects of genetic variation on the structure and maintenance of the hair follicle and pilosebaceous unit. Two novel loci, 11q12.2 and 17q25.3, contain genes encoding key enzymes involved in lipid biosynthesis pathways.

## Introduction

Acne vulgaris (acne) is a common skin disorder characterized by comedones and inflammatory lesions (papules, pustules, nodules and cysts), especially on the face, neck, chest, and back [1, 2]. Acne affects about 85% of adolescents and young adults [3]. It usually regresses by the age of 25 years, but persistence into adulthood is significant, with about 26% of women and 12% of men reporting acne in their 40s [4]. Acne severity varies widely, from mild to moderate up to severe forms in 10% of adolescents as well as young adults [1]. Irreversible facial scarring occurs in up to 20% of teenagers, with a significant long-term negative impact on emotional and mental health including low self-esteem and social isolation. Higher rates of anxiety, depression with suicidal ideation, and an elevated rate of unemployment make acne a significant social and health care burden [5, 6].

The pathogenesis of acne is multifactorial, involving an interplay of several factors [5]. Last decade has set a focus on abnormal differentiation of progenitor cells of the hair follicle (HF) that generate the sebaceous glands (SG) and infundibulum: the “comedo switch“ [7]. These leucine-rich repeat and immunoglobulin-like domain 1 positive (LRIG1+) cells in the junctional zone (JZ) of the pilosebaceous unit (PSU) can potentially differentiate into infundibular or SG duct/SG cells. Dysfunctional progenitors (inability to migrate, abnormal cell division and lack of differentiation) will result in cells remaining in the junctional zone, causing it to expand, thus giving rise to comedones. Due to the defect in cellular motility, the SGs are not replenished and will become atrophic [8, 9]. The pathogenesis of acne inflammation is still not fully understood, although certain *C. acnes* phylotypes (IA1) or lack of microbial diversity might trigger the responses from immune system [10].

Twin studies have shown that acne is highly heritable, with up to 85% of the population variance attributed to genetic factors [11]. Cross-sectional prevalence studies suggest a similar genetic contribution, with the family history correlating strongly with the disease risk and severity [2]. Concordant with current knowledge of the pathogenesis of acne, genetic studies have outlined biological pathways with overlapping functions, such as stem cell homeostasis, tissue remodeling, cell adhesion, and androgen metabolism [12–14]. Genome-wide association studies (GWASs) have led to the identification of 46 susceptibility loci in European populations, highlighting genes with established roles in the determination of hair follicle development, morphology, and activity and underscoring the importance of TGF-β–mediated signaling pathways [15–18]. GWASs and studies of single-gene disorders and syndromes associated with acne have revealed strong links to the tissue microenvironment and remodeling, occurring during sebaceous gland maturation, but not to inflammation, which thus may be considered a secondary event [12]. Understanding the biological processes that govern SG development and homeostasis would reveal potential etiological mechanisms underlying acne and other SG-associated skin disorders [8].

Yet, a significant proportion of the genetic susceptibility to acne remains unclear; therefore, conducting comparative studies with different populations is essential. The replication of previous findings is crucial to identify true risk loci and unravel key trait-related pathways. We carried out a GWAS with the Estonian Biobank (EstBB) cohort data (30,194 cases, 94,694 controls), followed by a meta-analysis of data from three independent cohorts (34,422 affected individuals and 364,991 population controls) of European ancestry.

## Materials and methods

### Study cohorts

Data were collected from the EstBB, FinnGen, and Lifelines study cohorts. The reporting of acne cases varied among the cohorts and consisted of clinical assessment documentation, retrieval of ICD-10 codes from electronic health records, and self-reported diagnoses. The final sample consisted of 34,422 cases and 364,991 controls. Detailed information regarding informed consent, ethical approval, recruitment, and the definition of acne for each cohort is provided in Supplementary File 1.

### Genome-wide association analysis and meta-analysis

Genome-wide single markers were tested using a mixed-effects logistic regression model. Data from the EstBB and FinnGen cohorts were analyzed using REGENIE v2.0.2 [19], and those from the Lifelines cohort were analyzed using SAIGE v0.43.1 [20]. Sex and the first 10 PCs were included as covariates, the latter to reduce the confounding effect introduced by the population structure. Age and genotyping batch were additionally included as covariates for the FinnGen cohort.

Prior to the meta-analysis, all variants were aligned to positions on human genome build GRCh37 (hg19), and variants with imputation INFO score <0.4 and minor allele frequency <1% were excluded. An inverse-variance fixed-effects meta-analysis of data from the three cohorts was performed with genomic control correction using METAL (version 2011-03-25) [21]. Variants present in all three cohorts (total = 6,438,535) were retained for downstream analysis.

### Functional annotation of acne risk loci

We used independent computational pipelines for the functional annotation of the GWAS meta-analysis results. Newly identified significant genome-wide risk loci were defined by FUnctional Mapping and Annotation of Genome-Wide Association Studies (FUMA) v1.3.7 [22] SNP2GENE tool (using positional, expression quantitative trait loci (eQTL) and chromatin interaction mapping) as non-overlapping genomic regions with a linkage disequilibrium (LD) window (*r*^2^ ≥ 0.6) around each lead variant with *P*_Meta_ < 5 × 10^−8^. Association signals were merged into a single genomic locus if the distance between lead variants was less than 250 kb. We used Multi-marker Analysis of GenoMic Annotation (MAGMA) v1.08 [23], implemented in FUMA v1.3.7, for gene-based association (we applied a Bonferroni corrected significance of 2.53 × 10^−6^ (0.05/19,785 genes tested)), and gene-set analysis. Genes defined by SNP2GENE were tested with the GENE2FUNC tool in FUMA for the examination of differentially expressed genes in GTEx v8 tissue types and overrepresentation in various gene sets. Default values were set for multiple testing correction (the Benjamini–Hochberg method), the adjusted *P* value cutoff (0.05), and the minimum number [2] of overlapping genes. In addition, the functional impact of variants of interest was explored by directly querying available eQTL associations in skin samples (non-sun-exposed and sun-exposed skin) using the GTEx v8 database (https://www.gtexportal.org).

### Heritability estimation

The proportion of variance in acne liability explained by common single nucleotide polymorphisms (*h*^2^_SNP_) was estimated using LDSC v1.0.1 [24]. HapMap phase 3 SNPs (excluding the HLA region) and precomputed LD scores from 1000 Genomes phase 3 data from European-ancestry populations were used. Heritability estimates were reported on a liability scale, assuming a 30% population prevalence of acne.

### Polygenic risk score calculation

Polygenic risk scores (PRSs) were calculated using PRS-CS-auto (version 2021-01-04) [25], which infers posterior SNP effect sizes under continuous shrinkage priors based on GWAS summary statistics and an external LD reference panel consisting of 503 European-ancestry population samples in 1000 Genomes dataset. For PRS calculation, stratified sampling of EstBB study participants for sex and reported acne conglobata cases (ICD-10 L70.1) was performed to split the data into training and validation sets in a 2:1 ratio (training set contains 20,130 cases and 63,130 controls; validation set contains non-overlapping 10,064 cases and 31,564 controls). The polygenicity of acne susceptibility was assessed by a PRS leveraging genome-wide effects across all SNPs.

Each PRS was standardized, and average PRSs for acne vs. control groups were compared using the two-tailed Student’s *t*-test. The PRSs were categorized by percentiles (<5, 5–10, 10–15, 15–20, 20–80, 80–85, 85–90, 90–95, and ≥95%), and the risk for each category was estimated relative to the average risk (20–80%) using logistic regression adjusted for sex and the first 10 PCs.

## Results

### Meta-analysis results

**Fig. 1:**
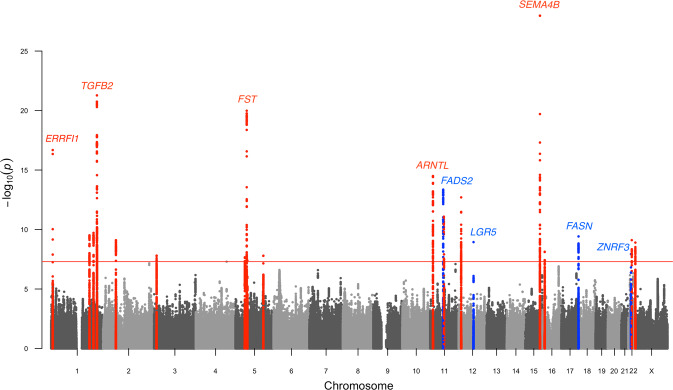
Manhattan plot from the meta-analysis of 34,423 acne vulgaris cases and 377,643 controls in three European-ancestry cohorts. The *x*-axis shows the genomic position (chromosomes 1–22 and X) and the *y*-axis shows the −log_10_ association *P* values for SNPs and indels. The red line indicates the genome-wide significance threshold of 5 × 10^–8^. Novel significant loci are in blue (*n* = 4) and previously identified significant loci are in red (*n* = 19).

Study-level Manhattan and Q–Q plots of three population-based cohorts (EstBB, FinnGen, and Lifelines) are presented in Figs. S1, S2. The results from the 34,422 acne cases and 364,991 population controls were combined using meta-analysis (Fig. 1). There was no evidence of excessive genomic inflation (λ_Meta_ = 1.033) in the GWAS meta-analysis (LD score regression intercept = 0.940 (SE = 0.008)) (Fig. S2D). Association summary statistics attaining genome-wide significance (*P* < 5 × 10^−8^) in fixed-effects meta-analysis together with study-level association summary statistics are presented in Table S1. All common SNPs across the genome explained 9.4% (SE = 0.0095) of the phenotypic variance (SNP-based heritability) in acne liability.

We identified 1230 significant genome-wide associations (*P*_Meta_ < 5 × 10^−8^), which were mapped to 23 risk loci (Table 1); four loci (11q12.2, 12q21.1, 17q25.3, and 22q12.1) were novel. The association peaks on chromosomes 11, 17, and 22 encompassed several genes, but were ascribed through fatty acid desaturase 2 (*FADS2*), fatty acid synthase (*FASN*), and zinc and ring finger 3 (*ZNRF3*), respectively, after gene prioritization. Of the previously identified risk loci, we replicated 19 at the genome-wide significance level, and seven were borderline significant (*P* < 10^−6^, Table S2).

**Table 1:** Genome-wide significant associations with acne vulgaris.

Chr	Pos (GRCh37)	RA	PA	rsID	Band	RA Freq	OR (95% CI)	*P* value	Gene region
1	8207579	C	G	rs80293268	1p36.23	0.080	1.17 (1.13–1.21)	2.10 × 10^−17^	*ERRFI1-(SLC45A1)*
1	183166970	T	C	rs3118181	1q25.3	0.509	1.06 (1.04–1.08)	3.08 × 10^−10^	*LAMC2*
1	202358947	T	C	rs2696958	1q32.1	0.499	1.06 (1.04–1.08)	1.93 × 10^−10^	*PPP1R12B-UBE2T-LGR6*
1	218858745	A	G	rs1481362	1q41	0.418	1.10 (1.08–1.12)	5.33 × 10^−22^	*TGFB2*
1	219199380	C	G	rs1256580	1q41	0.141	1.11 (1.08–1.14)	2.71 × 10^−15^	*LYPLAL1-DT*
2	60520351	T	A	rs9808396	2p16.1	0.443	1.06 (1.04–1.08)	7.88 × 10^−10^	*BCL11A*
3	12030034	T	C	rs2600262	3p25.2	0.787	1.07 (1.04–1.09)	1.57 × 10^−8^	*TIMP4-PPARG*
5	44362134	C	T	rs10941664	5p12	0.714	1.06 (1.04–1.08)	2.18 × 10^−8^	*FGF10*
5	52628130	C	T	rs37776	5q11.2	0.354	1.10 (1.08–1.12)	1.04 × 10^−20^	*ITGA1-ITGA2-MOCS2-FST*
5	55596919	A	G	rs185094	5q11.2	0.068	1.11 (1.07–1.15)	4.01 × 10^−8^	*ANKRD55*
5	131797578	T	C	rs2522051	5q31.1	0.458	1.05 (1.04–1.07)	1.59 × 10^−8^	*SLC22A5-IRF1-IL5-(RAD50)*
11	13130559	T	C	rs11022666	11p15.3	0.349	1.08 (1.06–1.10)	3.19 × 10^−15^	*RASSF10-ARNTL*
**11**	**61619829**	**C**	**A**	**rs174594**	**11q12.2**	**0.379**	**1.08** (**1.06**–**1.10)**	**4.45** × **10**^**−14**^	***FADS1-FADS2***
11	64611543	C	T	rs11231890	11q13.1	0.797	1.08 (1.06–1.11)	8.33 × 10^−12^	*CDC42BPG*
11	65521126	G	T	rs11227289	11q13.1	0.438	1.06 (1.04–1.08)	1.23 × 10^−8^	*OVOL1-MAP3K11*
12	12604777	A	G	rs10734852	12p13.2	0.538	1.07 (1.05–1.09)	1.99 × 10^−13^	*BORCS5*
**12**	**71691648**	**A**	**G**	**rs4760791**	**12q21.1**	**0.586**	**1.06** (**1.04**–**1.08)**	**1.14** × **10**^**−9**^	***TSPAN8-LGR5***
15	90734426	C	T	rs34560261	15q26.1	0.839	1.16 (1.13–1.19)	1.06 × 10^−28^	*SEMA4B*
16	11034933	G	A	rs6498135	16p13.13	0.593	1.06 (1.04–1.08)	7.39 × 10^−9^	*CIITA-DEXI-CLEC16A*
**17**	**80117478**	**C**	**A**	**rs8078102**	**17q25.3**	**0.242**	**1.07** (**1.05**–**1.09)**	**3.82** × **10**^**−10**^	***FASN-CCDC57-SLC16A3***
**22**	**29453193**	**G**	**C**	**rs12321**	**22q12.1**	**0.641**	**1.06** (**1.04**–**1.08)**	**1.15** × **10**^**−8**^	***ZNRF3-C22orf31-KREMEN1***
22	33221375	G	A	rs130291	22q12.3	0.546	1.06 (1.04–1.08)	7.83 × 10^−10^	*TIMP3*
22	50321928	C	T	rs7410766	22q13.33	0.215	1.07 (1.05–1.10)	1.26 × 10^−9^	*ALG12-CRELD2-PIM3*

ORs represent the risk of a risk allele relative to a protective allele. Novel loci are in bold.

*Chr* chromosome, *Pos* position, *RA* risk allele, *PA* protective allele, *RA Freq* frequency of risk allele, *OR* odds ratio, *CI* confidence interval.

### Functional annotation and gene prioritization results

**Fig. 2:**
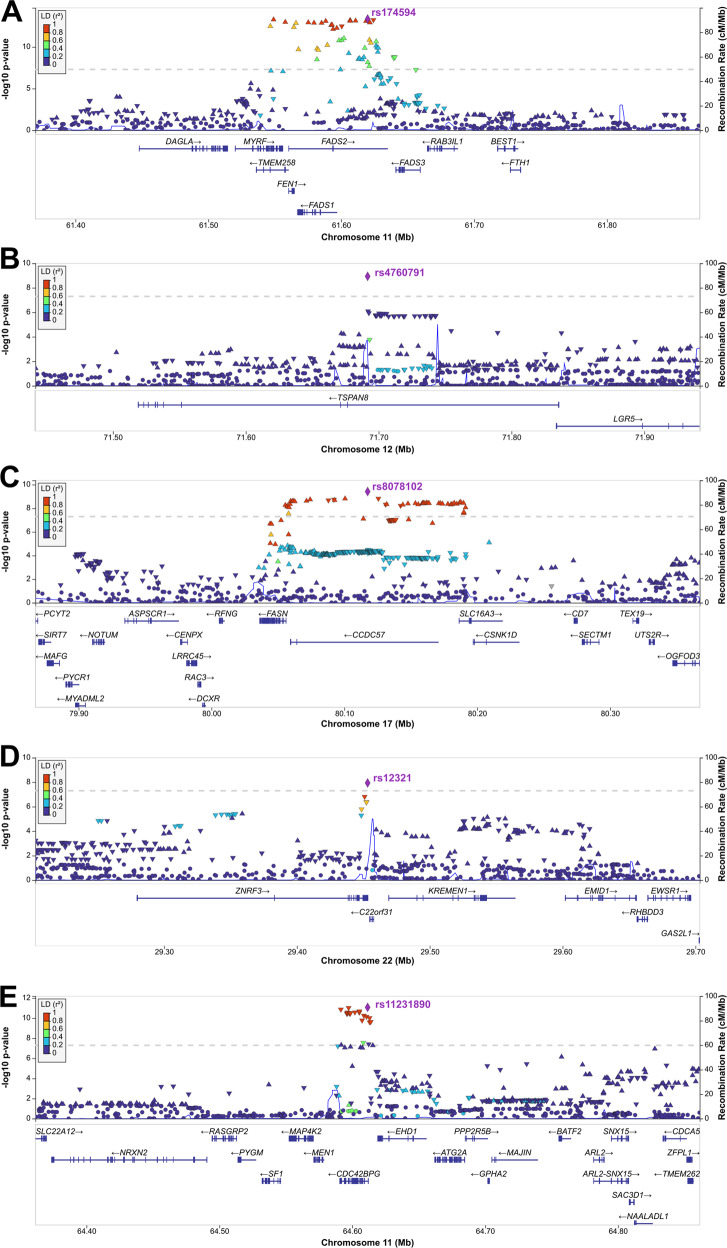
Regional association plots for novel acne vulgaris susceptibility loci and the known locus 11q13.1 with a second independent association signal for rs11231890 from the GWAS meta-analysis data. (**A**) 11q12.2, rs174594; (**B**) 12q21.1, rs4760791; (**C**) 17q25.3, rs8078102; (**D**) 22q12.1, rs12321; (**E**) 11q13.1, rs11231890. SNPs are color coded to reflect their LD (*r*^2^) with the lead SNP on each panel.

The 11q12.2 locus (chr11:61542006–61656117, GRCh37) with the lead variant rs174594 (OR = 1.08, 95% CI 1.06–1.10, *P* = 4.45 × 10^−14^) in *FADS2* intron 5 encompasses fatty acid desaturase 1 (*FADS1*)*, FADS2*, and transmembrane protein 258 (*TMEM258*) genes, all significantly associated by FUMA positional, eQTL, and/or chromatin interaction mapping (Table S3, Figs. 2A, S3A) and highlighted as genome-wide significant genes by MAGMA gene-based testing (Fig. S4, Table S5). *FADS1* and *FADS2* are expressed in SG cells of sun-exposed skin (Fig. S5). Moreover, eQTL analysis revealed significantly increased *FADS2* and *TMEM258* expression in sun-exposed and non-sun-exposed skin linked to the risk alleles of the 11q12.2 credible set SNPs (Table S4).

The 12q21.1 locus is defined by the independent lead variant rs4760791 (OR = 1.06, 95% CI 1.04–1.08, *P* = 1.14 × 10^−9^) in tetraspanin 8 (*TSPAN8*) intron 2, with a CADD score of 19.2 and RegulomeDB score of 3a (Table S3, Figs. 2B, S3B). eQTL analysis revealed significantly reduced expression of the neighboring leucine-rich G protein–coupled receptor 5 (*LGR5*) gene in sun-exposed skin tissue, defined by the rs4760791 risk allele (*P* = 1.4 × 10^−5^, FDR = 7.15 × 10^−4^, Fig. S6). Multi-tissue eQTL comparison for *LGR5* and rs4760791 in GTEx revealed that sun-exposed skin was the only tissue predicted to show an eQTL effect of rs4760791 on *LGR5* expression (Fig. S7).

The 17q25.3 locus (chr17:80044078–80191189, GRCh37) with the lead variant rs8078102 (OR = 1.07, 95% CI 1.05–1.09, *P* = 3.82 × 10^−10^) in the intron of the coiled-coil domain containing 57 (*CCDC57*) (Table S3, Figs. 2C, S3C) also encompasses the *FASN* and solute carrier family 16 member 3 (*SLC16A3*) genes in this region; only *FASN* showed genome-wide significance in gene-based testing (*P* = 1.2 × 10^−7^; Fig. S4, Table S5). *FASN* and *CCDC57* are highly expressed in skin cell types, including SG cells, adipocytes, and mature keratinocytes (Fig. S8).

The 22q12.1 locus (chr22:29448643-29455687, GRCh37) harbors rs12321 (OR = 1.06, 95% CI 1.04–1.08, *P* = 1.14 × 10^−9^) in the 3’UTR of E3 ubiquitin-protein ligase Zinc and ring finger 3 (*ZNRF3*), with a CADD score of 21.7 (Table S3, Figs. 2D, S3D). *ZNRF3* and the neighboring kringle containing transmembrane protein 1 (*KREMEN1*) are expressed in several types of skin cells, including sebocytes and basal, suprabasal and mature keratinocytes (Fig. S9).

Evidence for a second independent association signal at the 11q13.1 locus was observed for rs11231890 (OR = 1.08, 95% CI 1.06–1.11, *P* = 8.33 × 10^−12^) in the intron of the CDC42 binding protein kinase gamma (*CDC42BPG*) (Table S3, Figs. 2E, S10). Conditional analysis for rs11231890 in the EstBB dataset confirmed the independence of its effect from rs11227289 (~900 kb downstream) in known acne locus *OVOL1*–*MAP3K11* (*P* = 4.07 × 10^−11^). Likewise, MAGMA gene-based testing supported the genome-wide significance of the *CDC42BPG* (*P* = 1.78 × 10^−11^; Fig. S4, Table S5), which was strongly expressed in sun-exposed and non-sun-exposed bulk tissue samples, particularly in basal keratinocytes (Fig. S11). The risk allele of rs11231890 significantly increased the expression of this gene in sun-exposed skin (FDR = 1.33 × 10^−8^; Table S4).

In addition, we report seven previously known acne loci with borderline genome-wide significant associations (*P* < 10^−6^; Table S2), with the strongest signals observed for 2q35 (rs146599639, *P* = 6.99 × 10^−8^ and rs72966077, *P* = 9.53 × 10^−8^), identifying the Wnt family member 10A (*WNT10A*), and 4q31.22 (rs72955609, *P* = 5.06 × 10^−8^), highlighting the endothelin receptor type A (*EDNRA*) (Table S2).

Evidence supporting the prioritization of genes from four novel genome-wide significant loci, and the known locus 11q13.1 with a second independent association signal, is presented in Table S9.

MAGMA gene-based analysis led to the identification of 50 genes with genome-wide significance (*P* < 2.53 × 10^−6^) after Bonferroni correction (Table S5). Subsequent competitive gene-set analysis demonstrated enrichment in several possibly relevant gene ontology (GO) biological processes: “GO branch elongation of an epithelium,” “GO regulation of metalloendopeptidase activity,” and “GO regulation of the trail-activated apoptotic signaling pathway”. For Curated gene sets, “KEGG melanoma” and the “Biocarta TCR pathway” were identified (Table S6).

A set of 323 genes prioritized by the SNP2GENE tool and showing expression in skin was selected for further analysis with the GENE2FUNC tool. Analysis against differentially expressed genes in 54 tissue types in the GTEx v8 RNA-seq data demonstrated their significant upregulation in sun-exposed skin and downregulation in thyroid, liver, testis, and several brain areas (Fig. S12). Among GO biological processes, the most significant gene-set enrichment occurred for “GO regulation of steroid biosynthetic process”, “GO regulation of lipid metabolic/biosynthetic process” and “GO steroid biosynthetic process”. Among other pathways, “Reactome regulation of cholesterol biosynthesis by SREBP-SREBF”, “Reactome activation of gene expression by SREBF-SREBP”, “Reactome signaling by wnt”, “Hallmark cholesterol homeostasis”, and “Hallmark MTORC1 signaling” were most significant. The enrichment of the prioritized genes in biological pathways and functional categories is presented in Table S7.

### Polygenic prediction of acne risk

**Fig. 3:**
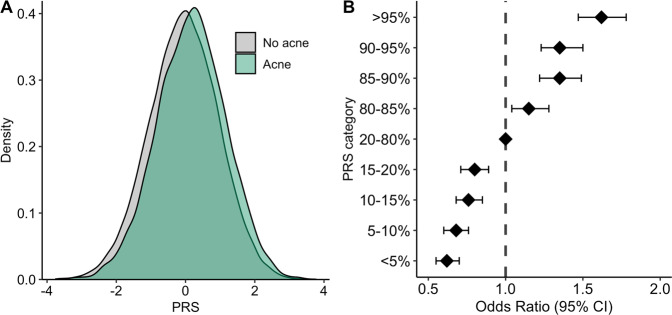
Acne PRS analysis. (**A**) Distributions of acne PRSs in individuals with no acne (gray) and acne (green). (**B**) Associations between categorized PRSs (<5, 5–10, 10–15, 15–20, 20–80, 80–85, 85–90, 90–95, ≥95%) and acne risk relative to the average risk (20–80%).

The polygenicity of acne susceptibility in the EstBB cohort was assessed using PRSs, with the leveraging of genome-wide effects across all variants using the PRS-CS-auto algorithm. Individuals reporting acne had significantly higher mean acne PRSs than did those from the control group (*t-*test, *P* = 6.87 × 10^−82^; Fig. 3A).

In the validation set, we observed significant risk stratification between percentiles. Individuals in the top 5% of the PRS distribution had a risk of acne development elevated by 1.62 (95% CI 1.47–1.78) times and those in the 90–95% PRS stratum had a risk increased by 1.35-fold (95% CI 1.23–1.50) relative to individuals with average risk (20–80%; Fig. 3B, Table S8).

## Discussion

Our meta-analysis identified four novel loci and brought the number of acne susceptibility loci in European populations to 50. We replicated 19 loci at the genome-wide significance level and seven at borderline significance level (*P* < 10^−6^). Therefore, 20 of the known 46 susceptibility loci in Europeans did not replicate. Likewise other studies in populations of European descent [15–18], we found no evidence of association at the two loci observed in the Han Chinese population for severe disease [26]. Common variants across the genome (SNP-based heritability) account for 9.4% of the phenotypic variance, indicating that additional loci contributing to disease susceptibility remain to be discovered.

In recent years acne has been reframed as a disorder of SG progenitor differentiation and migration [8]. SG development is coupled to HF morphogenesis, which relies on an extensive epithelial-mesenchymal crosstalk, involving Wnt, EDAR, Bmp, Hedgehog and FGF signaling pathways [27]. Canonical Wnt signaling supports morphogenesis of HF [28] by promoting different lineage choices in specific pilosebaceous stem cell populations [29, 30]. Activation of Wnt signaling in JZ LRIG1+ cells results in expansion of the upper HF [29] and downregulation of Wnt signaling promotes keratin 15-expressing bulge cells to migrate and differentiate into sebocytes [30]. Imbalances in Wnt pathway may lead to abnormal fate determination of the LRIG1+ stem cells, promoting the growth of the JZ instead of SG and leaving the lesion-associated SGs atrophic. Thus, comedones are most likely formed by abnormal differentiation of infundibular keratinocytes, which may become hyperproliferative [8]. Interestingly, Wnt signaling can be modulated by aryl hydrocarbon receptor (AHR) activation [31] in response to increased androgen synthesis at the onset of puberty [1]. By some evidence *C. acnes* can directly activate AHR signaling and induce terminal differentiation of sebocytes into keratinocyte-like cells [32], but there’s no data to prove if this applies to sebaceous progenitors [10].

Three novel acne associated genes are also involved in the Wnt signaling pathway: *ZNRF3* and *KREMEN1* from 22q12.1 and *LGR5* from 12q21.1. Previous GWAS implicated two genes from the same pathway - *LGR6* and *WNT10A* [17].

ZNRF3 and ring finger protein 43 (RNF43) are negative regulators of Wnt/β-catenin signaling cascade, mediating the ubiquitination, endocytosis, and subsequent degradation of frizzled (FZD) Wnt receptor complex components, resulting in the inhibition of canonical and non-canonical Wnt/catenin signaling pathways. *ZNRF3* and the neighboring *KREMEN1* are both expressed in SG cells and basal, suprabasal and differentiated keratinocytes derived from interfollicular epidermal stem cells [28]. The Dickkopf-1 (DKK1), KREMEN1 and low-density-lipoprotein receptor-related protein 6 (LRP6) complex is recognized as a major ligand-receptor complex regulating Wnt signaling in vertebrates [33]. The DKK1 context-dependent activity of KREMEN1 can inhibit or increase Wnt/β-catenin signaling by removal of or binding to LRP6 [34]. By promoting the turnover of Wnt receptors, FZD, LRP5/6, and ZNRF3 (also RNF43) ensure the proper levels of Wnt activity in stem cells [35]. Loss-of-function mutations in *ZNRF3* (and *RNF43*) potentiate the Wnt/β-catenin signaling cascade and β-catenin-independent Wnt-signaling cascades [36].

LGR5, a surface-expressed marker of adult stem cell populations in HF, participates in the maintenance and regulation of Wnt signaling via its association with Wnt receptors and mediating Wnt agonists (R-spondins, ligands for LGR5) [9, 37]. In mice, Lgr5 and Lgr6 exhibit restricted, yet non-overlapping expression patterns in resting adult hair follicles: Lgr5+ cells contribute progeny to all hair follicle components (excluding the sebaceous gland), whereas Lgr6+ cells contribute exclusively differentiated progeny to the sebaceous gland and interfollicular epidermis over entire lifetime [37]. Lgr5 expression occurs in stem cells at sites of active proliferation, being the direct consequence of active Wnt signaling [38], e.g., activation of Wnt signaling in HF LGR5+ bulge cells promotes hair growth [29]. Wnt signaling potentiation can take place through formation of the ZNRF3/RNF43–RSPO–LGR4/5/6 tertiary complex, resulting in the accumulation of FZD receptors on the plasma membrane and corresponding enhanced response to the Wnt ligands in cells [39].

Novel acne susceptibility gene *TSPAN8* is a member of the transmembrane 4 (tetraspanin) superfamily. Tetraspanins are implicated in processes relying on cell migration, e.g., cancer invasion, wound healing, and inflammation [40]. *TSPAN8* was shown to function as a negative regulator of integrin-linked kinase driven β1 integrin-dependent adhesion in melanoma cells, thereby decreasing adhesive interaction with the surrounding matrix environment and promoting tumor invasiveness [41]. Downregulation of *TSPAN8* was associated with IL-1β mediated inhibition of the Akt/MAPK pathway [42].The analysis of differentially expressed genes in 18 acne lesions and 18 paired normal skin samples from two datasets led to the identification of *TSPAN8* as one of the few genes concurrently down-regulated in acne samples [43]. We detected significant enrichment of *TSPAN8*, along with other novel genes (*LGR5, ZNRF3, KREMEN1*) and several known acne susceptibility genes, for the GO biological processes gene sets “GO negative regulation of response to stimulus” and “GO regulation of response to stress”.

Two novel acne associated loci, identified in this study, contain genes encoding enzymes involved in lipid metabolism: FADS1/FADS2 and FASN. Although acne lesion-associated SGs are atrophic, sebum production in general is abnormally high and suppression of sebum secretion can reduce acne incidence and severity [44]. The composition of sebum differs from that in normal skin by higher levels of monounsaturated fatty acids (MUFA) and unbalanced ratio of triglycerides to wax esters [45]. Recent study presented significantly higher induction of sebum-type MUFAs with chain length of 14–17 carbon atoms at both forehead and cheek areas in acne patients [46].

Remarkably, human sebum production is highly dependent on local flux through the de novo lipogenesis (DNL) pathway and is >20% higher in acne patients [47]. Up to 85% of the two major fatty acids (FA) in human sebum, palmitate and sapienate (a non-essential FA exclusive to human sebum), is derived from DNL, while the contribution of DNL to circulating very-low-density-lipoprotein (VLDL)-triglyceride palmitate is only ~20%. These findings demonstrate the major role of sebocyte DNL pathway in the overproduction of sebum lipids in humans with acne and highlight the inhibitors of DNL key enzymes as potential therapeutic agents [47]. Palmitic acid, a precursor of sapienic acid, is synthesized in the terminal catalytic steps of long-chain saturated FA synthesis pathway by the multifunctional metabolic enzyme FASN [48]. The level of FASN expression reflects stages of sebocyte differentiation. Along with high level expression of nuclear androgen receptor (AR), high levels of FASN and PPARγ are detected in middle-stage differentiated cells, whilst late-stage differentiating sebocytes, in the upper part of the gland, exhibit low levels of AR, FASN, and PPARγ and are positive for B-lymphocyte induced maturation protein (BLIMP1/PRDM1), marker of terminal differentiation in all epidermal lineages [49].

The rate-limiting enzyme FADS2 can desaturate at least ten substrates: eight polyunsaturated fatty acids (PUFA), one MUFA when PUFAs are low, and one saturate, palmitic acid into sapienic acid, the predominant unsaturate in human skin [50]. The enhanced DNL in acne may cause a prominent decrease in synthesis of anti-inflammatory PUFA molecules like EPA, DHA and DGLA, because palmitic acid competes with linoleic and α-linolenic acids for FADS2-mediated Δ6-desaturation [50]. Genetic variants in *FADS2* cluster were shown to be associated with 18C-PUFA to LC-PUFA conversion efficiencies [51]. The risk-increasing C allele of our top SNP rs174594 in *FADS2* is in high LD (*r*^2^ = 0.89) with rs3834458 delT allele in the promoter region. The carriage of delT allele has been associated with decreased activity of rate-limiting FADS2 enzyme, manifesting as lower levels of EPA, DPA and DHA in the blood compared to those of major allele homozygotes. Thus, variations in *FADS2* may influence the rate of conversion of α-linolenic acid into other n-3 PUFAs [52]. As today’s western diet contains a low ratio of n-3 to n-6 FAs, this will deepen the low levels of anti-inflammatory EPA and DHA content in delT carriers.

EPA and especially DHA, can inhibit dimerization of TLR-1 and TLR-2 [53] and consequent activation of TLR-signaling pathways in keratinocytes and macrophages in response to *C. acnes*, which otherwise leads to hyperproliferation of keratinocytes and the initiation of inflammatory reaction. Expression of keratinocyte TLR-2 and TLR-4 leads to activation of NF-κB and MAPK pathways and subsequent production of IL-1, IL-6, IL-8, TNF-α, human beta-defensin-2, granulocyte-macrophage colony-stimulating factor (GM-CSF), and matrix metalloproteinases (MMP) [54]. Activation of monocyte TLR-2 leads to the production of proinflammatory cytokines, including IL-1, TNF-α, IL-8, and IL-12 [55]. Thus, DHA and EPA, inhibiting activation of TLR-signaling pathways, may decrease the inflammatory response in patients with acne.

Based on GWAS data from 2/3 of randomized EstBB study participants, we developed an acne PRS that demonstrated strong associations with reported acne status and allows better acne risk stratification. Additional studies including more cases are needed to develop an acne PRS with enhanced predictive utility. Clarification of the roles of genetic factors underlying the observed phenotypic variability will provide a better understanding of the etiology of acne and may eventually enable the earlier identification of individuals at greater risk in combination with clinical risk factors; it will also highlight therapeutically actionable pathways that can be targeted for effective treatment options.

The current study ascertained acne diagnosis in EstBB cohort mostly by ICD-10 codes, but cases from Lifelines were all self-reported. Acne phenotype was not stratified by severity. It is probable that the meta-analysis cohort (especially EstBB) contains relatively more mild cases; however, some mild cases could remain underdiagnosed and may be included among controls. This may explain why this study lacked sufficient power to replicate approximately half of the known susceptibility loci and provided smaller heritability estimates compared to previously reported estimates.

In conclusion, this study provides additional insight into the genetic predisposition for acne. The identification of novel acne susceptibility genes belonging to Wnt-signaling pathway, known as a determinant of stem and progenitor cell differentiation during hair follicle morphogenesis and regeneration, emphasizes the imbalanced SG homeostasis in acne pathogenesis. The detection of specific loci, containing genes, which encode key enzymes in lipid metabolism, emphasizes the conception of acne, being etiologically linked to other metabolic diseases. Further studies with more diverse populations are warranted to discover more risk loci and to elucidate the biological processes and pathways that mediate the genetic risk of acne.

### Supplementary information

Supplementary File 1

Table S1

Table S2

Table S3

Table S4

Table S5

Table S6

Table S7

Table S8

Table S9

## Data Availability

Meta-analysis summary statistics generated during the current study have been deposited in the GWAS Catalog (https://www.ebi.ac.uk/gwas/) with accession number GCST90245818. The individual level data from Estonian Biobank are available under restricted access and can be obtained after permission of the Estonian Committee on Bioethics and Human Research.
