# Correction: Genome-wide meta-analysis identifies novel loci conferring risk of acne vulgaris

**DOI:** 10.1038/s41431-023-01366-0

**Published:** 2023-04-20

**Authors:** Maris Teder-Laving, Mart Kals, Anu Reigo, Riin Ehin, Telver Objärtel, Mariliis Vaht, Tiit Nikopensius, Andres Metspalu, Külli Kingo

**Affiliations:** 1 Estonian Genome Center, Institute of Genomics, University of Tartu, Tartu, Estonia; 2 Institute for Molecular Medicine Finland (FIMM), HiLIFE, University of Helsinki, Helsinki, Finland; 3 Institute of Health Technologies, Tallinn University of Technology, Tallinn, Estonia; 4 BioCC Ltd, Tartu, Estonia; 5 Institute of Molecular and Cell Biology, University of Tartu, Tartu, Estonia; 6 Faculty of Medicine, Institute of Clinical Medicine, University of Tartu, Tartu, Estonia; 7 Tartu University Hospital, Tartu, Estonia

**Keywords:** Genome-wide association studies, Genetics research

Correction to: *European Journal of Human Genetics* 10.1038/s41431-023-01326-8, published online 16 March 2023

In Fig. 3B of this article *y*-axis is labelled in reverse order; Fig. 3 should have appeared as shown below.

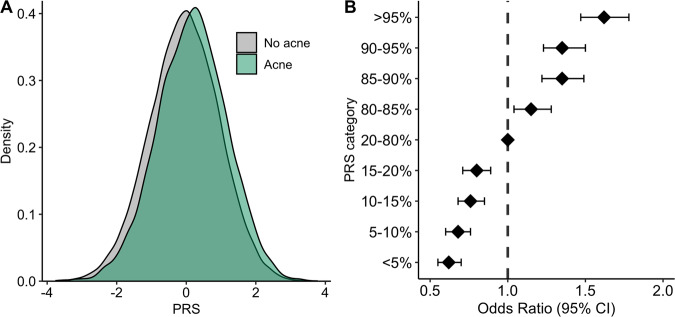



The original article has been corrected.

