# Supplementary material for: Genome-wide meta-analysis identifies novel loci conferring risk of acne vulgaris
Source: Eur J Hum Genet. 2023 Mar 16;32(9):1136–43. doi: 10.1038/s41431-023-01326-8 (PMC11368920; doi:10.1038/s41431-023-01326-8)

Supplementary information

Supplementary Data File 1

# **Cohort details**

Estonian Biobank

The Estonian Biobank (EstBB) is a population-based biobank with more than 200,000 participants (https://genomics.ut.ee/en/content/estonian-biobank). The EstBB project is being conducted according to the Estonian Gene Research Act, and all participants have signed a broad informed consent form (1). Acne cases (*n* = 30,193, current mean age 34.44 (SD = 9.53) years, 74.5% females) were defined by the ICD-10 codes L70.0 (acne vulgaris), L70.1 (acne conglobata), L70.5 (acne excoriata), L70.8 (other acne), L70.9 (acne, unspecified). Data were collected from the biobank’s health database which is regularly linked with national electronic health registries (including hospital databases). In addition, upon recruitment participants completed a questionnaire, including data about their health status, lifestyle and diet - 3.7% of acne cases (1,121 out of 30,193) are assigned only on the basis of mentioned medically assisted questionnaire and the rest 96.3% of acne diagnosis are coming from the questionnaire and/or electronic health records. EstBB participants with no diagnosis of L70, L71, or L73 served as controls (*n* = 94,694, current mean age 40.32 (SD = 9.42) years, 60.0% females). All individuals are of European descent.

FinnGen

FinnGen is a public–private partnership with the aim of collecting and analyzing genome and health data from 500,000 Finnish biobank participants (https://www.finngen.fi/en). Patients and control subjects in the FinnGen provided informed consent for biobank research, based on the Finnish Biobank Act. Clinical endpoints and controls are defined by the FinnGen clinical expert groups - acne endpoint is defined by the ICD-10 code L70 from the hospital discharge and cause of death registries. The present study includes 1,558 acne cases (mean age at first event 27.96 years, 57.7% females) and 250,988 controls from Data Freeze 6. Individuals with disorders of skin appendages defined by ICD-10 codes L60-L75 were excluded from controls. All individuals are of European descent.

Lifelines

Lifelines is a multidisciplinary prospective population-based cohort study with a unique three-generation design that examines the health and health-related behaviors of 167,729 participants living in the northern Netherlands (2,3). In Lifelines, adult cohort acne cases were ascertained by their positive answer to the question if they have (had) severe acne (in years 2007-2013) and if they have (had) serious acne complaints (in years 2019-2023). In the youth cohort (13-17 years) acne was ascertained by their positive answer (“yes, a little”, “yes, a lot”, “yes, extremely”) to the question if they have pimples and how much they suffered from this problem in the last year. Overall, 2,671 cases (current mean age 31.32 (SD = 14.67) years, 56.7% females) and 19,309 controls (current mean age 41.87 (SD = 9.13) years, 60.1% females) from the Lifelines sample were examined. All individuals are of European descent.

# **Genotyping and imputation**

Estonian Biobank

The EstBB samples were genotyped at the Core Genotyping Lab of the Institute of Genomics, University of Tartu, using Illumina global screening arrays v1.0, v2.0 and v2.0_EST. Individuals whose sex defined based on X chromosome heterozygosity did not match the sex recorded in phenotype data were excluded from the analysis. Before imputation, variants were filtered by call rate < 95%, Hardy–Weinberg equilibrium *P* < 1 × 10^-4^ (autosomal variants only), and minor allele frequency < 1%. Prephasing was performed using Eagle v2.3 software (4), and imputation was performed using Beagle v.28Sep18.793 (5) with the use of an Estonian population–specific imputation reference panel built from 2,297 whole-genome sequencing samples (6).

FinnGen

The FinnGen samples were genotyped with Illumina or Affymetrix (Thermo Fisher Scientific) arrays. Genotype calls were made with the GenCall and zCall algorithms for Illumina data, and the AxiomGT1 algorithm for Affymetrix data. Individuals with sex mismatches, high degrees (>5%) of genotype missingness, excess heterozygosity (±4 SD from the sample mean), and non-Finnish ancestry were excluded. In addition, variants with high degrees (>2%) of missingness, deviation from Hardy–Weinberg equilibrium (*P* < 1 × 10^-6^), and minor allele counts < 3 were excluded. Array-genotyped samples were pre-phased array-wise with Eagle v2.3.5. Genotype imputation was performed with Beagle v4.1 using a Finnish population–specific reference panel built from 3,775 high-coverage (25–30×) Finnish whole-genome sequencing samples.

Lifelines

The Lifelines samples were genotyped by the University Medical Center (Groningen) Genetics Lifelines Initiative with Illumina global screening array v1.0. Samples were excluded based on call rate < 97%, sex mismatches, and overall increased/decreased heterozygosity (±3 SD from the sample mean). Filtering was performed to exclude duplicate samples and sample mix-ups, and low-quality samples with call rates < 99% and excess heterozygosity (>4 SD from the mean). Possible genotyping errors were assessed at the marker level: variants with high degrees (>1%) of missingness, deviation from Hardy–Weinberg equilibrium (<1 × 10^-6^), and monomorphic markers were removed. Principal component analysis including genotype data from 1000 Genomes and Genome of the Netherlands project parental individuals were used to detect participants with non-European ancestry. Genotype imputation was performed through the Sanger imputation service using the Haplotype Reference Consortium panel v1.1 (7).

# **References**

1. Leitsalu L, Haller T, Esko T, Tammesoo ML, Alavere H, Snieder H, et al. Cohort Profile: Estonian Biobank of the Estonian Genome Center, University of Tartu. International Journal of Epidemiology. 2015 Aug 1;44(4):1137–47.

2. Klijs B, Scholtens S, Mandemakers JJ, Snieder H, Stolk RP, Smidt N. Representativeness of the LifeLines Cohort Study. PLOS ONE. 2015 Sep 2;10(9):e0137203.

3. Scholtens S, Smidt N, Swertz MA, Bakker SJ, Dotinga A, Vonk JM, et al. Cohort Profile: LifeLines, a three-generation cohort study and biobank. International Journal of Epidemiology. 2015 Aug 1;44(4):1172–80.

4. Loh PR, Danecek P, Palamara PF, Fuchsberger C, Reshef YA, Finucane HK, et al. Reference-based phasing using the Haplotype Reference Consortium panel. Nat Genet. 2016 Nov;48(11):1443–8.

5. Browning SR, Browning BL. Rapid and Accurate Haplotype Phasing and Missing-Data Inference for Whole-Genome Association Studies By Use of Localized Haplotype Clustering. Am J Hum Genet. 2007 Nov;81(5):1084–97.

6. Mitt M, Kals M, Pärn K, Gabriel SB, Lander ES, Palotie A, et al. Improved imputation accuracy of rare and low-frequency variants using population-specific high-coverage WGS-based imputation reference panel. Eur J Hum Genet. 2017 Jul;25(7):869–76.

7. McCarthy S, Das S, Kretzschmar W, Delaneau O, Wood AR, Teumer A, et al. A reference panel of 64,976 haplotypes for genotype imputation. Nat Genet. 2016 Oct;48(10):1279–83.

**Fig. S1.** Manhattan plots from GWASs for three cohorts: (A) EstBB (30,193 acne vulgaris cases and 94,694 controls), (B) FinnGen (1558 acne vulgaris cases and 250988 controls), and (C) Lifelines (2671 acne vulgaris cases and 19,309 controls). The *x*-axis shows the genomic position (chromosomes 1–22 and X) and the *y*-axis shows the –log_10_ (*P* value) of SNP association. The threshold for genome-wide significance was set at 5 × 10^−8^ (red horizontal line).

A


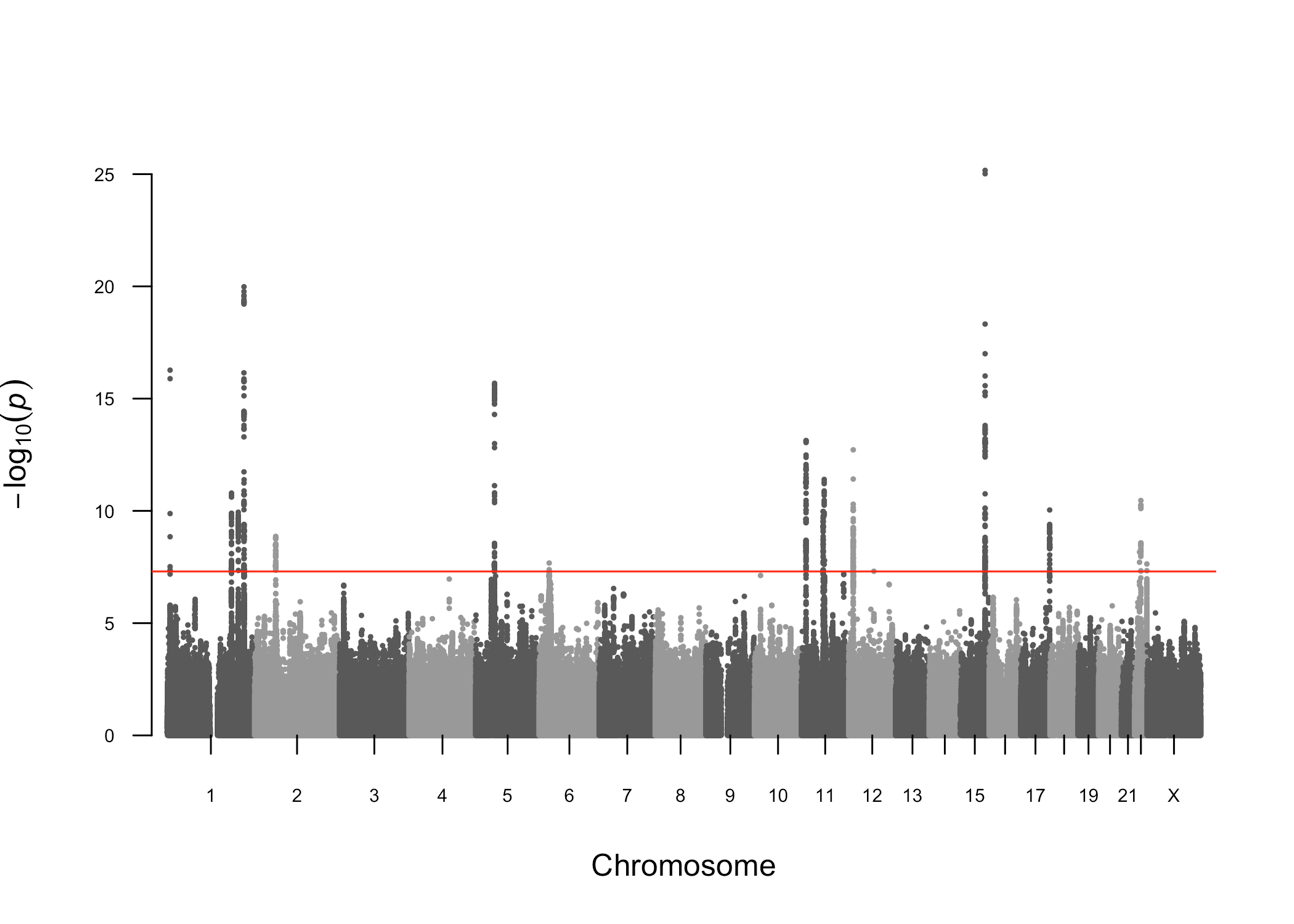


B


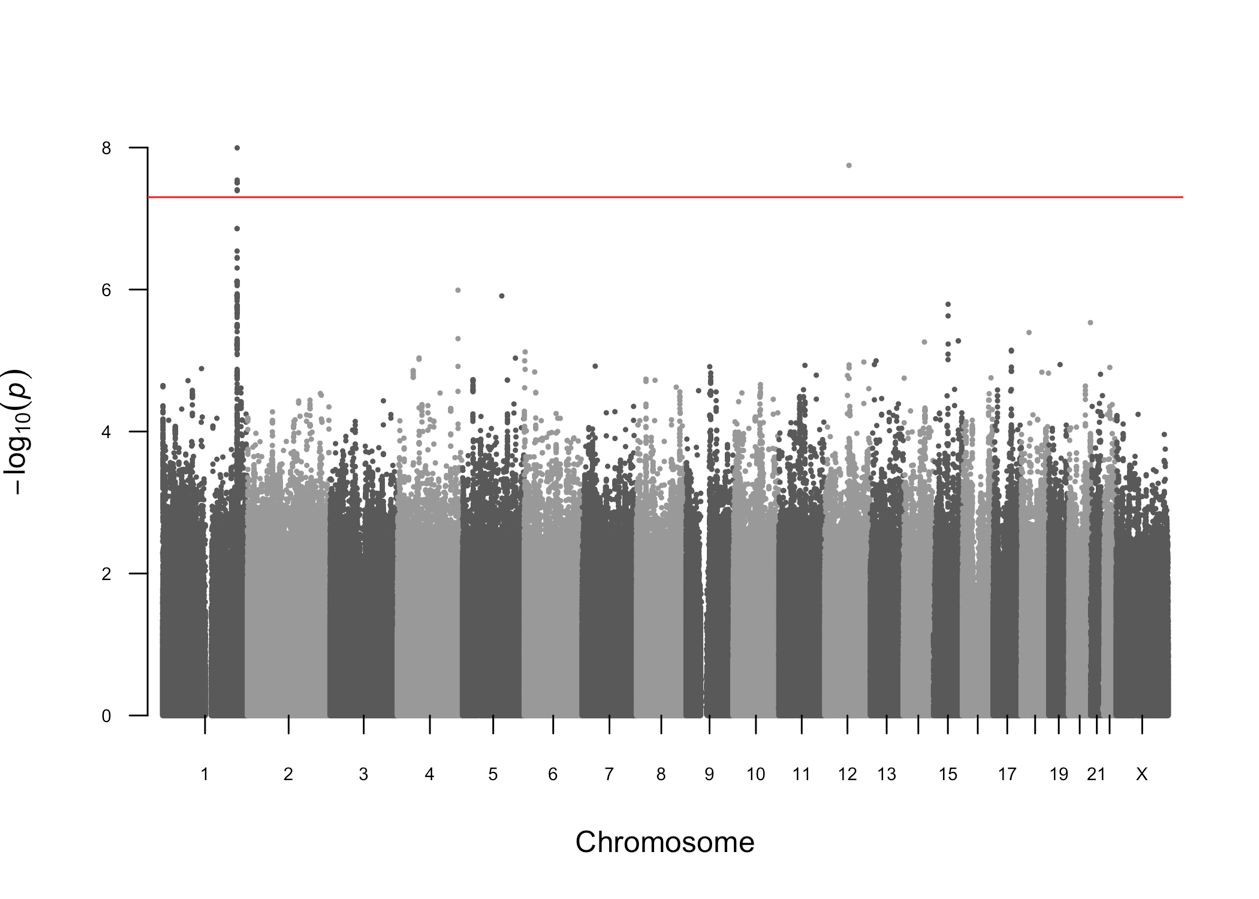


C


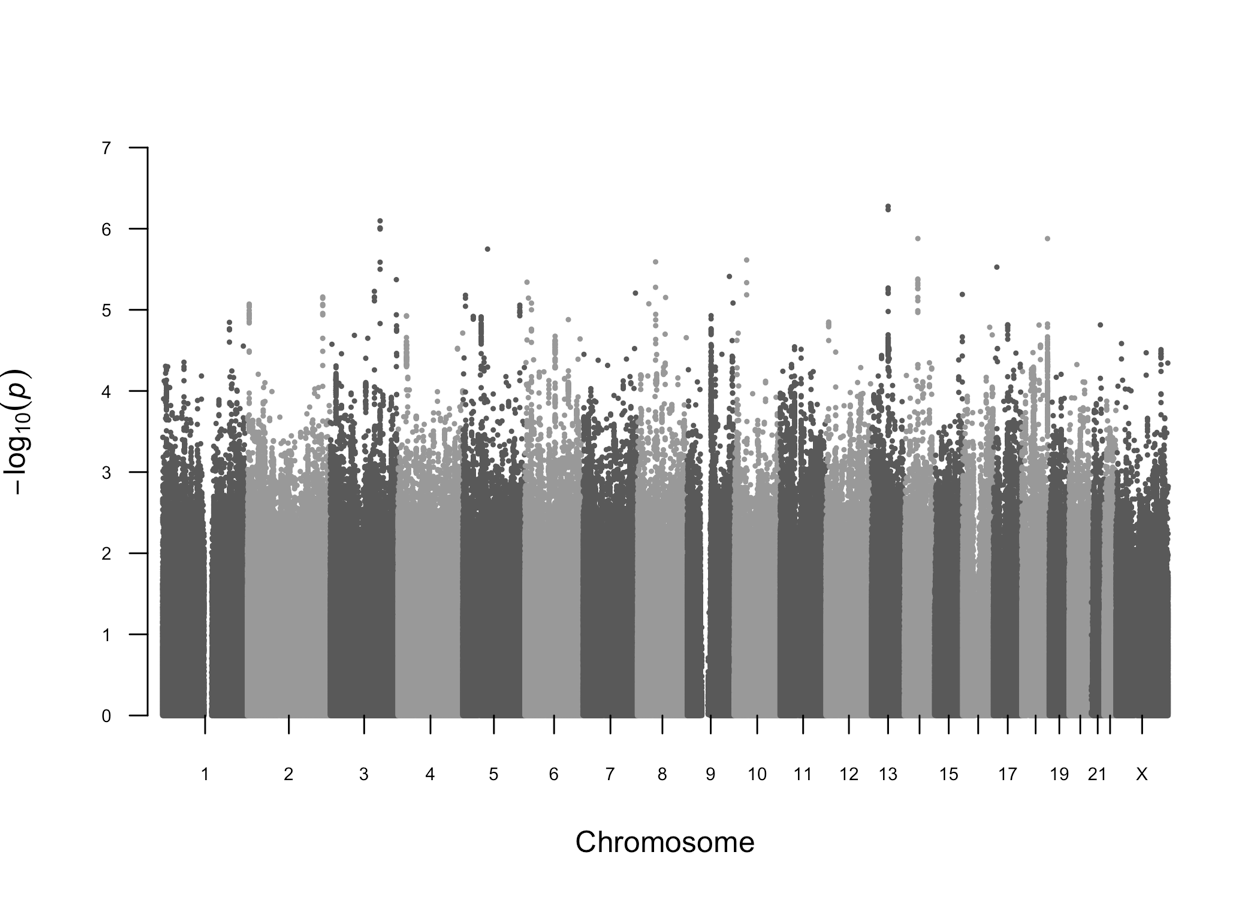


**Fig. S2**. Q-Q plots of GWAS data from the discovery cohorts: (A) EstBB (λ_EstBB_ = 1.140), (B) FinnGen (λ_FinnGen_ = 1.026), (C) Lifelines (λ_Lifelines_ = 1.003). (D) Meta-analysis results for 34,423 acne vulgaris cases and 377,643 controls from the three European ancestry cohorts (λ_Meta_ = 1.033).

A B

**
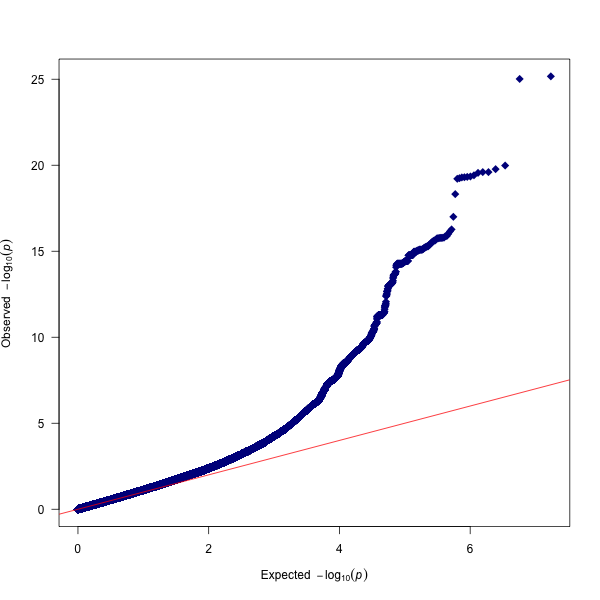

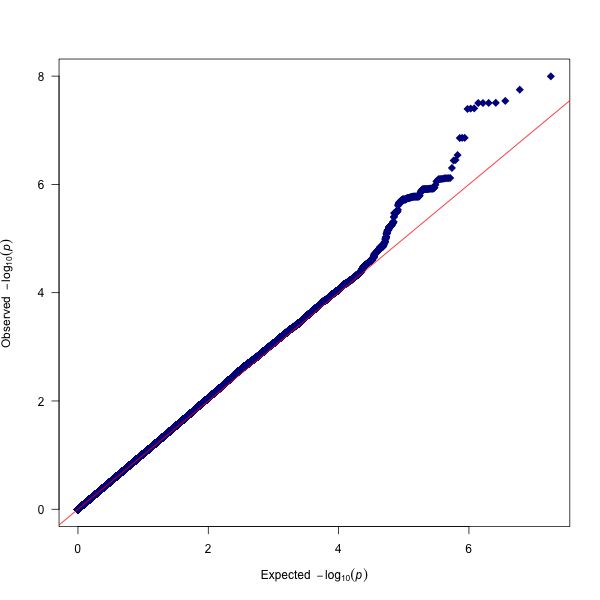
**

C D

**
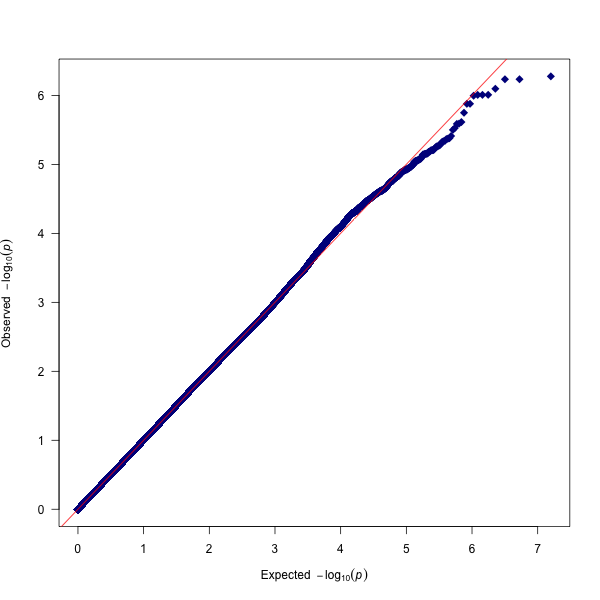

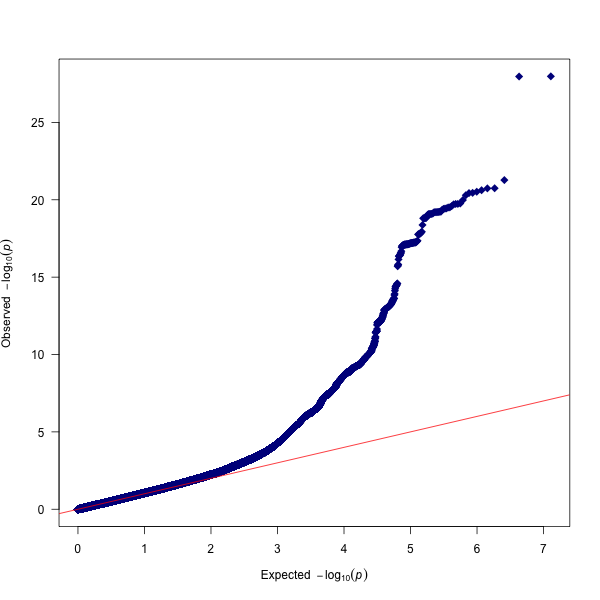
**

**Fig. S3.** Circos plots of novel loci for acne vulgaris. (A) 11q12.2, rs174594; (B) 12q21.1, rs4760791; (C) 17q25.3, rs8078102; (D) 22q12.1, rs12321. Outermost layer: Manhattan plot with the rsID of the top SNP. SNPs are color coded as a function of their maximum *r*^2^ values to the lead SNP in the locus: red, *r*^2^ > 0.8; orange, *r*^2^ > 0.6; green, *r*^2^ > 0.4; blue, *r*^2^ > 0.2; gray, *r*^2^ ≤ 0.2. The *y*-axis ranges from 0 to the maximum –log_10_ (*P* value) of the SNPs. Second layer: Chromosome ring with genomic risk loci shown in blue. Genes are mapped according to chromatin interactions (orange), eQTLs (green), or both (red). Third layer: chromosome ring without coordinates for the alignment of the positions of genes with genomic coordinates. Links are chromatin interactions (orange) and eQTLs (green).



**Fig. S4.** (A) Manhattan and (B) Q-Q plots of MAGMA gene-based testing. Input SNPs were mapped to 19,785 protein-coding genes. Genome-wide significance (red dashed line) was defined at *P* = 0.05/19,785 = 2.53 × 10^-6^. Gene names in red representing genes from new susceptibility loci.

A


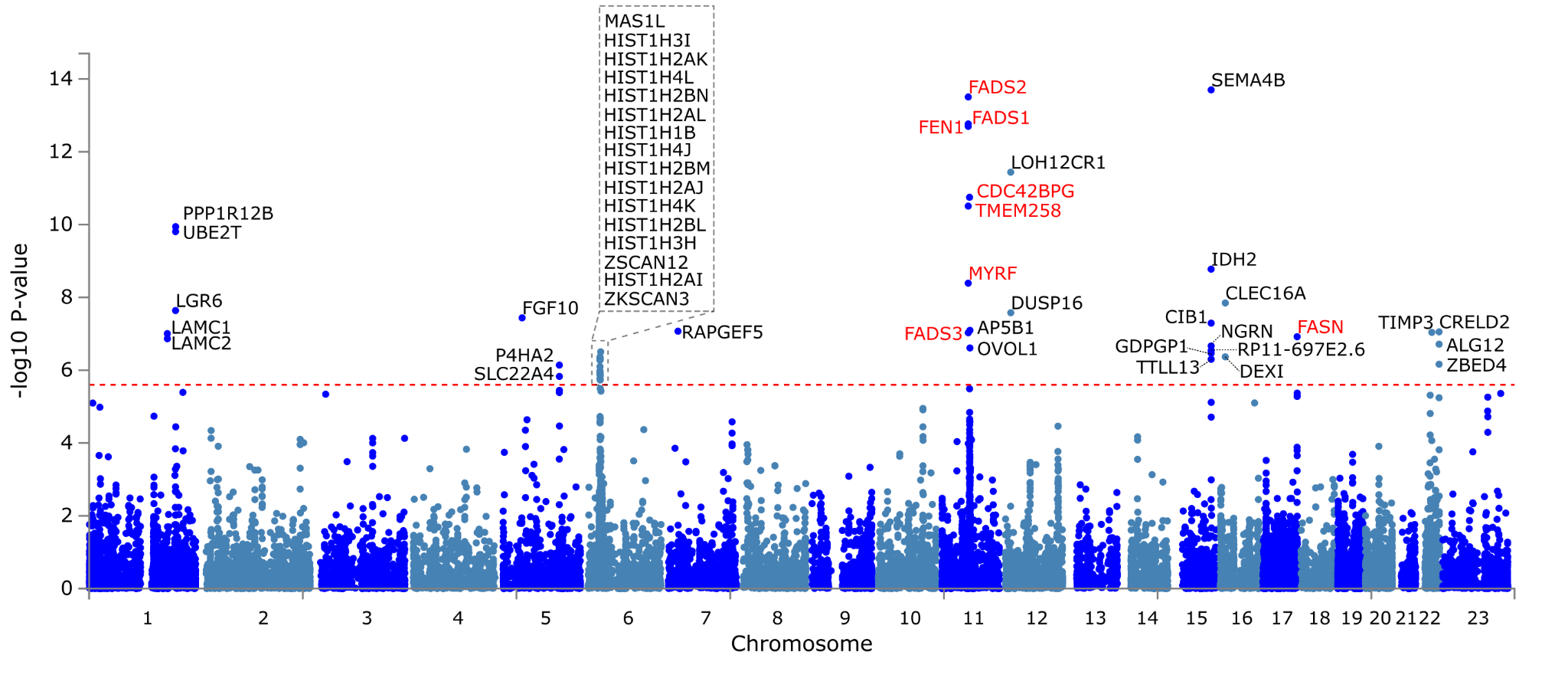


B


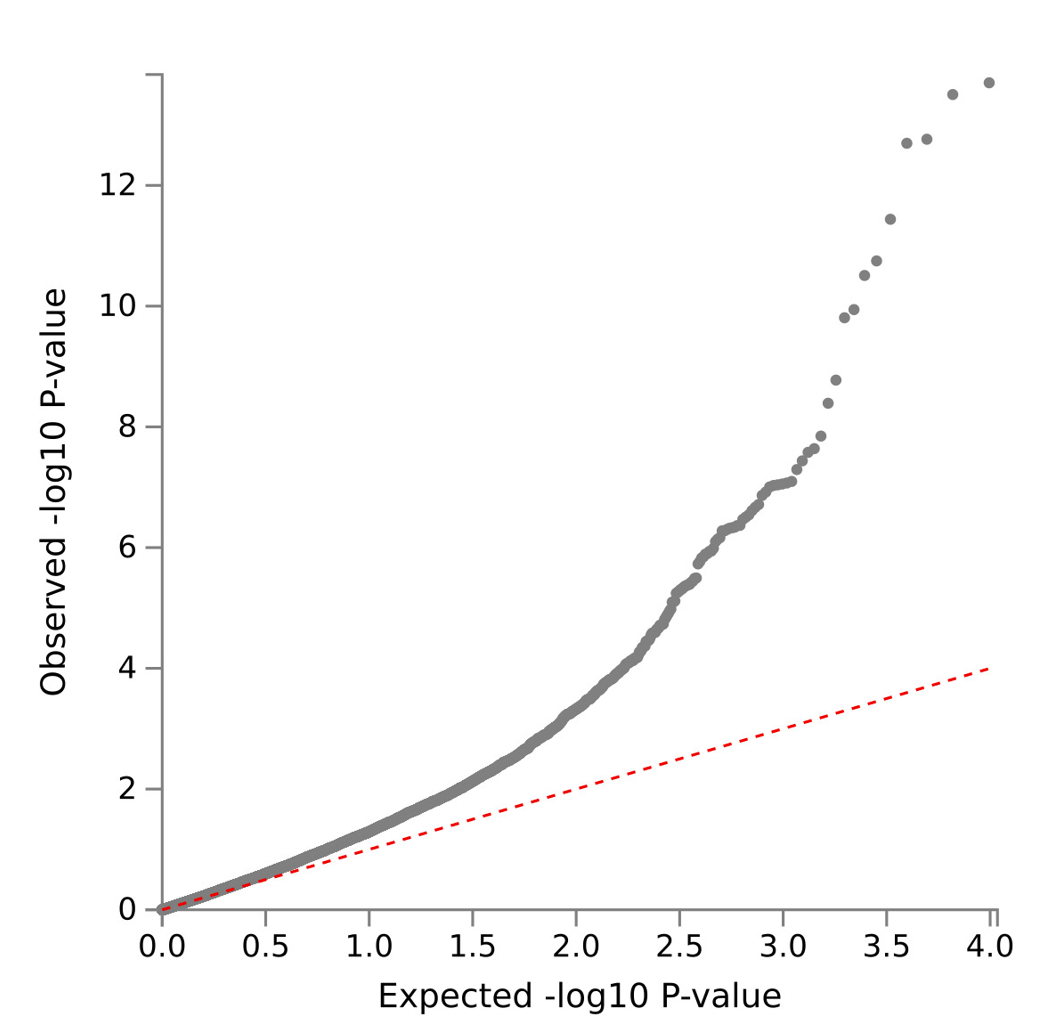


**Fig. S5.** Expression of (A) *FADS1* and (B) *FADS2* in sebaceous gland cells. Data source: GTEx project (gtexportal.org), single-cell snRNA-seq pilot. *Y*-axis: Median log(CP10K).


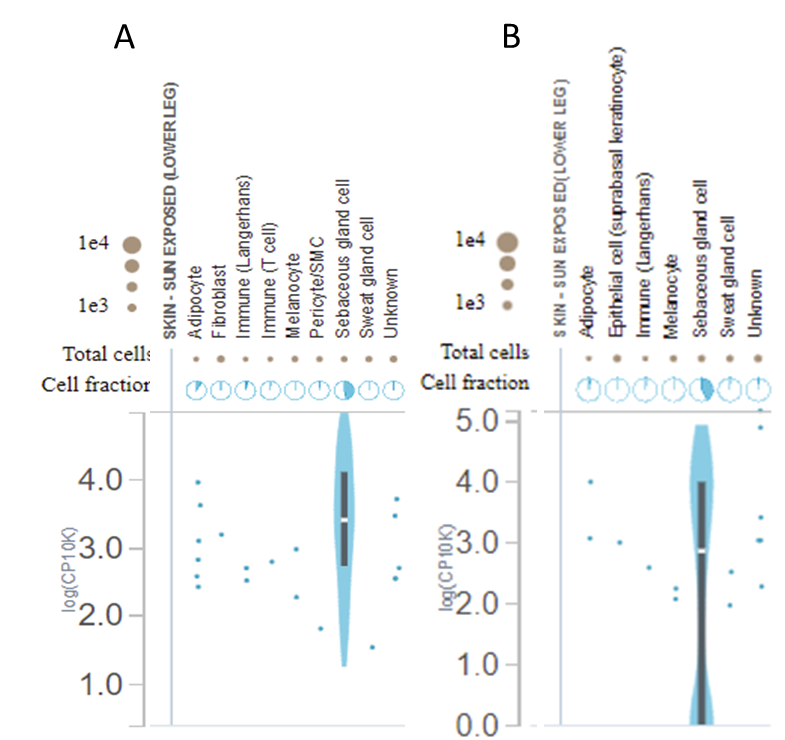


**Fig. S6.** Expression of *LGR5* in sun-exposed lower-leg skin, defined by rs4760791 genotypes AA, AG, and GG. Data was extracted from the GTEx project (gtexportal.org).


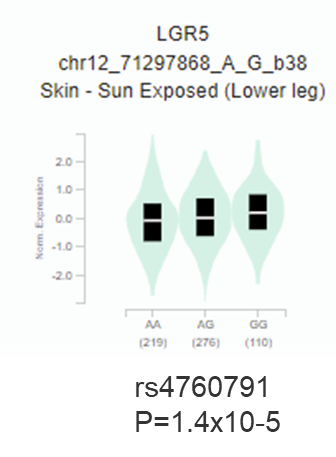


**Fig. S7**. Multi-tissue eQTL comparison for *LGR5* and rs4760791.

NES = eQTL effect size (the slope of the linear regression of normalized expression data vs. three genotype categories), determined by single-tissue analysis. The normalized expression values are based on quantile normalization for each tissue, followed by inverse quantile normalization for each gene across samples. m-value = posterior probability that an eQTL effect exists in each tissue tested in the cross-tissue meta-analysis. m-values > 0.9 signify that the tissue is predicted to have an eQTL effect and m-values < 0.1 signify that the tissue is predicted to have no eQTL effect; otherwise, the predicted existence of an eQTL effect is ambiguous. For sun-exposed skin eQTL p-value = 1.37 × 10^-5^, and MetaSoft m-value = 0.92. Data source: GTEx project (gtexportal.org).


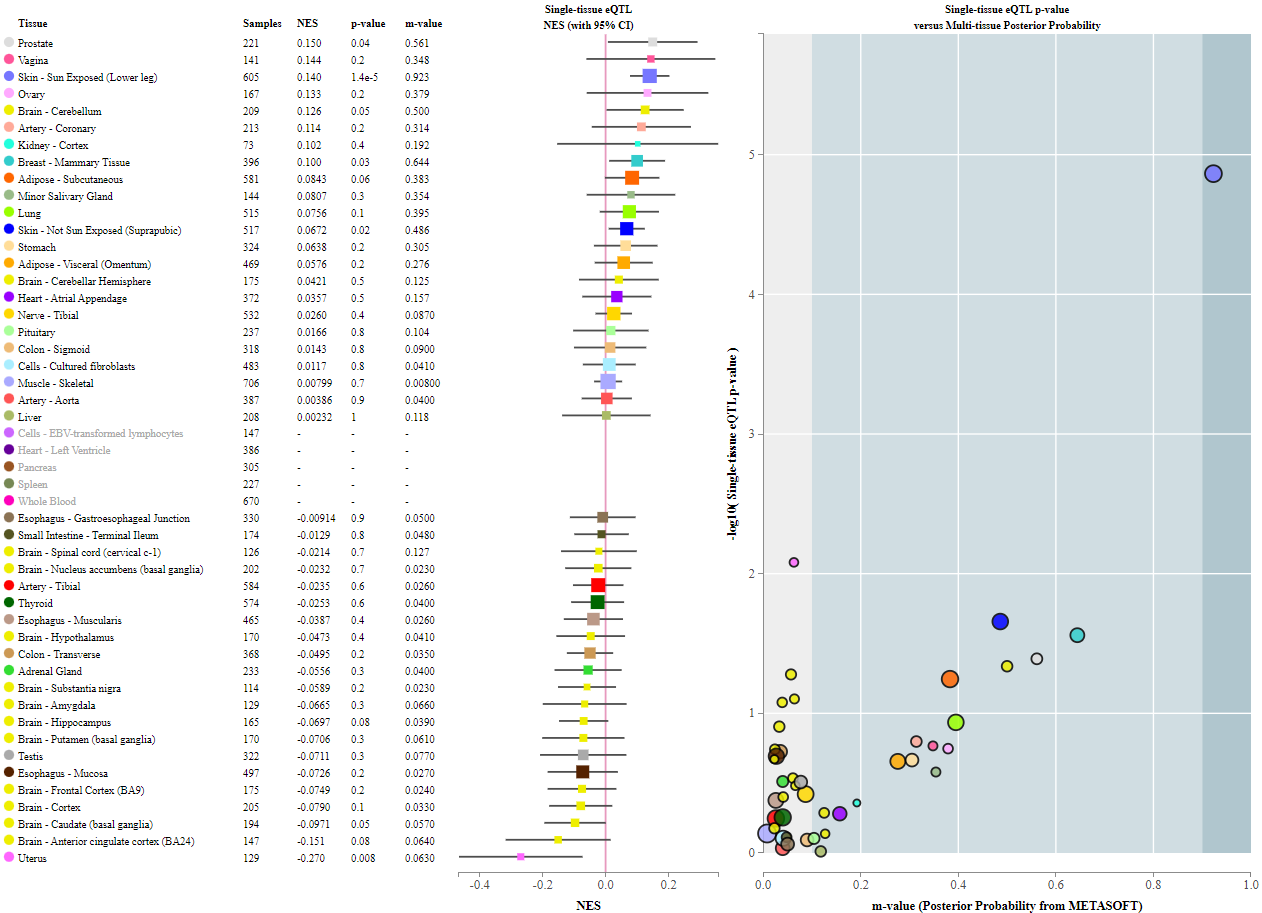


**Fig. S8.** Single tissue expression for *CCDC57* and *FASN*. Data source: GTEx project (gtexportal.org), single-cell snRNA-seq pilot. *Y*-axis: median log(CP10K).


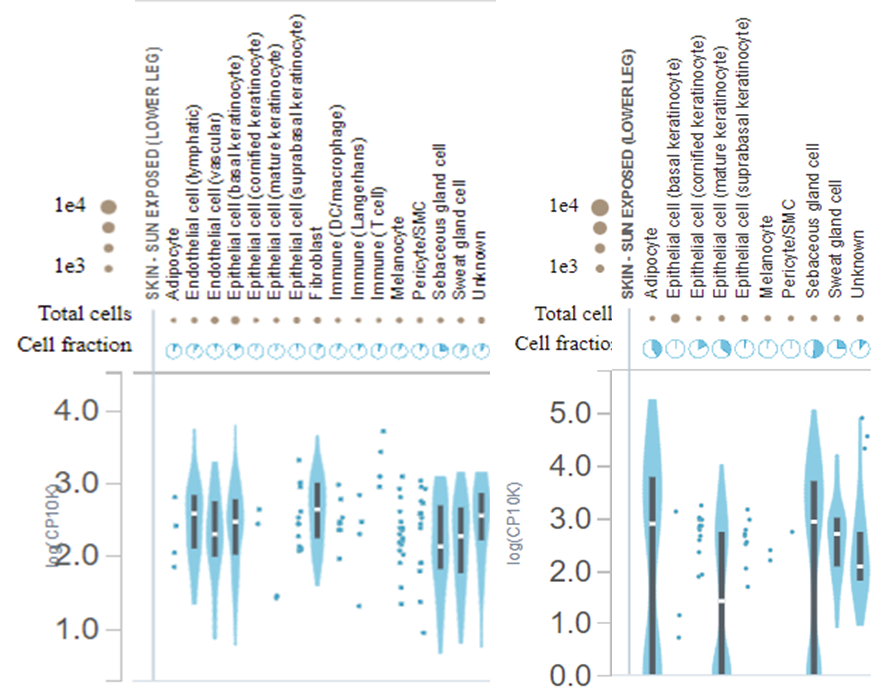


**Fig. S9.** Single-tissue expression for *ZNRF3* (A) and *KREMEN1* (B). Data source: GTEx portal (gtexportal.org), single-cell snRNA-seq pilot. *Y*-axis: median log(CP10K).


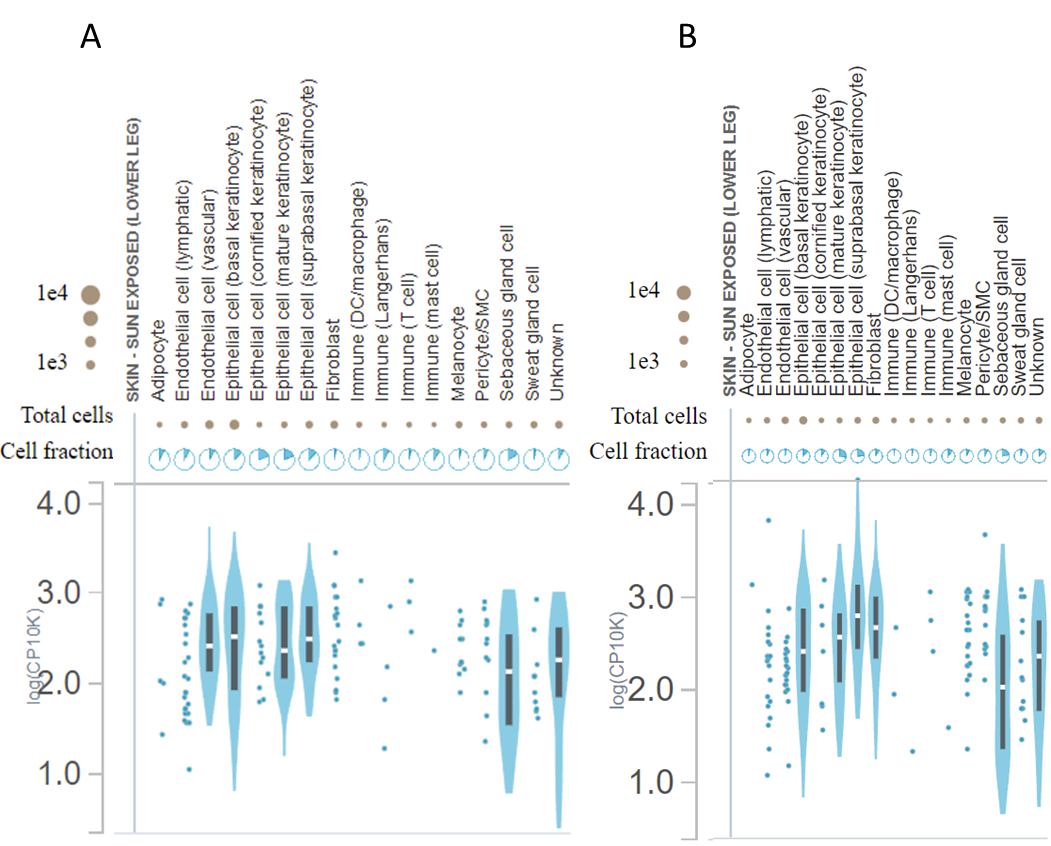


**Fig. S10.** Circos plot of known locus 11q13.1 with a second independent association signal for rs11231890, determined from GWAS and meta-analysis. Outermost layer: Manhattan plot with the rsID of the top SNP. SNPs are color coded as a function of their maximum *r*^2^ values to the lead SNP in the locus: red, *r*^2^ > 0.8; orange, *r*^2^ > 0.6; green, *r*^2^ > 0.4; blue, *r*^2^ > 0.2; gray, *r*^2^ ≤ 0.2. The *y* axis ranges from 0 to the maximum –log_10_ (*P* value) of the SNPs. Second layer: Chromosome ring with genomic risk loci shown in blue. Genes are mapped according to chromatin interactions (orange), eQTLs (green), or both (red). Third layer: chromosome ring without coordinates for the alignment of the positions of genes with genomic coordinates. Links are chromatin interactions (orange) and eQTLs (green).


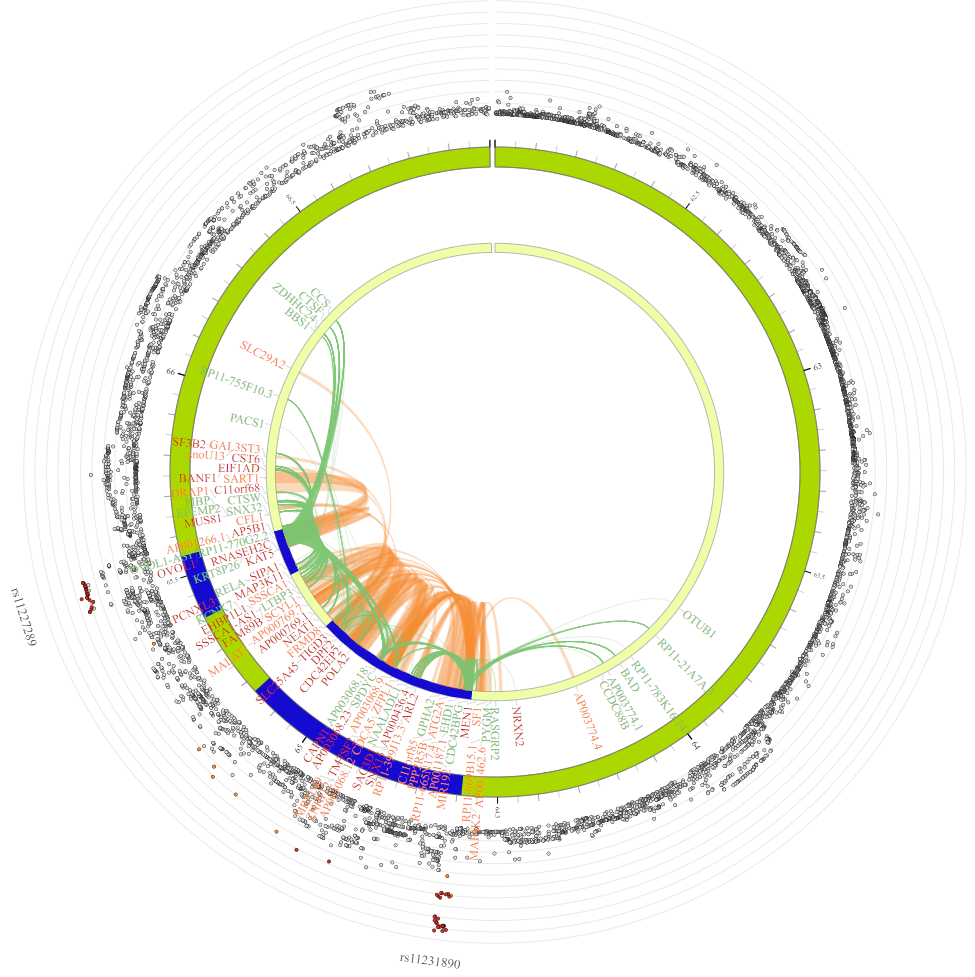


**Fig. S11.** Single-tissue expression for *CDC42BPG*. Data source: GTEx project (gtexportal.org), single-cell snRNA-seq pilot. *Y*-axis: median log(CP10K).


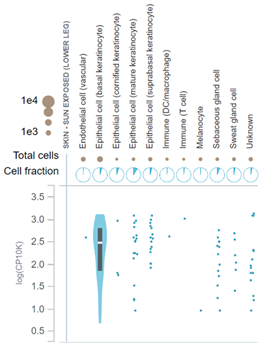


**Fig. S12.** Functional mapping and annotation GENE2FUNC differentially expressed gene (DEG) output, drawing on GTEx v8 data for 53 tissue types. Red bars denote significantly enriched DEG sets (*P* < 0.05, Bonferroni corrected).


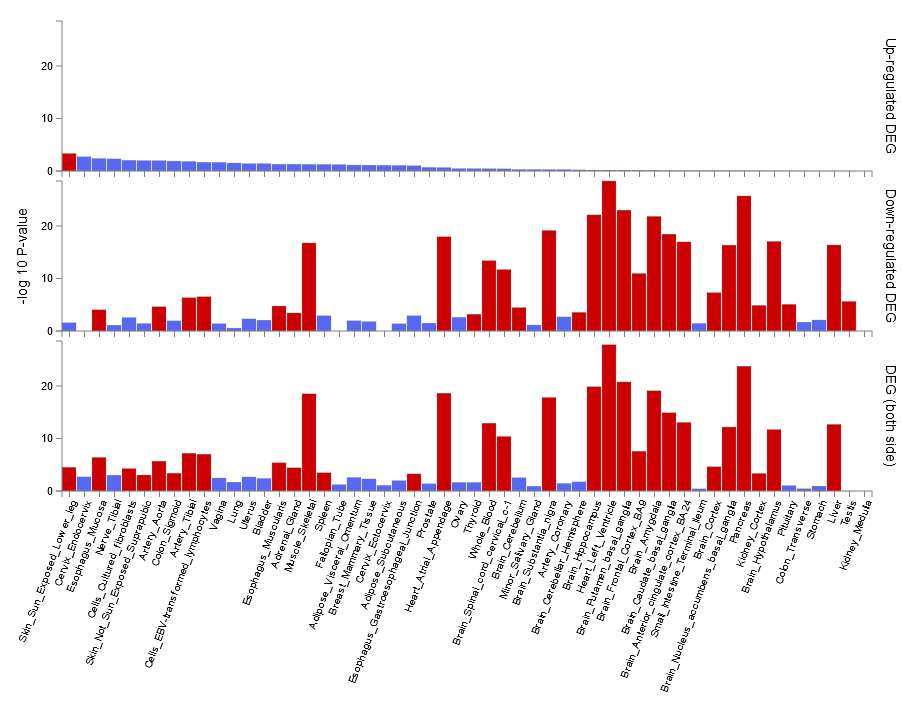

Supplement: Supplementary file 1 [file 41431_2023_1326_MOESM1_ESM.docx]
